# Chemical Tuning, Pressure, and Temperature Behavior of Mn^4+^ Photoluminescence in Ga_2_O_3_–Al_2_O_3_ Alloys

**DOI:** 10.1021/acs.inorgchem.2c02807

**Published:** 2022-11-01

**Authors:** Yaroslav Zhydachevskyy, Vitaliy Mykhaylyk, Vasyl Stasiv, Lev-Ivan Bulyk, Vasyl Hreb, Iryna Lutsyuk, Andriy Luchechko, Shusaku Hayama, Leonid Vasylechko, Andrzej Suchocki

**Affiliations:** † Institute of Physics, Polish Academy of Sciences, aleja Lotników 32/46, Warsaw02-668, Poland; ‡ Diamond Light Source, Harwell Campus, DidcotOX11 0DE, U.K.; § Lviv Polytechnic National University, South Bandera Street 12, Lviv79013, Ukraine; ∥ Ivan Franko National University of Lviv, Tarnavskogo Street 107, Lviv79017, Ukraine

## Abstract

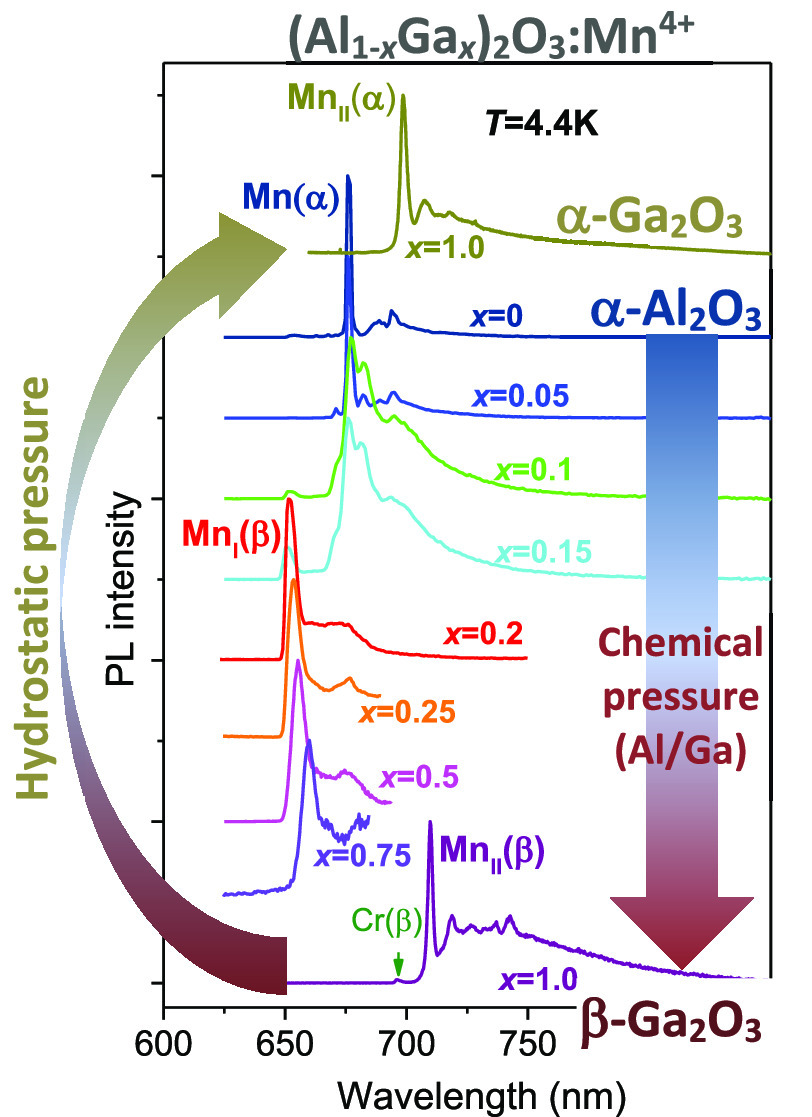

In this study, we carried out a detailed investigation of the photoluminescence of Mn^4+^ in Ga_2_O_3_–Al_2_O_3_ solid solutions as a function of the chemical composition, temperature, and hydrostatic pressure. For this purpose, a series of (Al_1–*x*_Ga_*x*_)_2_O_3_:Mn^4+^,Mg phosphors (*x* = 0, ..., 0.1.0) were synthesized and characterized for the first time. A detailed crystal structure analysis of the obtained materials was done by the powder X-ray diffraction technique. The results of the crystal structure and luminescence studies evidence the transformation of the ambient-pressure-synthesized material from the rhombohedral (α-type) to monoclinic (β-type) phase as the Ga content exceeds 15%. Spectroscopic features of the Mn^4+^ deep-red emission, including the temperature-dependent emission efficiency and decay time, as well as the possibility of their tuning through chemical pressure in each of these two phases were examined. Additionally, it has been shown that the application of hydrostatic pressure of ≥19 GPa allows one to obtain a corundum-like α-Ga_2_O_3_:Mn^4+^ phase. The luminescence properties of this material were compared with β-Ga_2_O_3_:Mn^4+^, which is normally synthesized at ambient pressure. Finally, we evaluated the possibility of application of the studied phosphor materials for low-temperature luminescence thermometry.

## Abstract 2

We carried out a detailed investigation of the photoluminescence of Mn^4+^ in Ga_2_O_3_−Al_2_O_3_ solid solutions as a function of the chemical composition, temperature, and hydrostatic pressure and evaluated the possibility of application of the studied phosphors for low-temperature luminescence thermometry.

## Introduction

Red-emitting Mn^4+^ -doped compounds belong to a very promising group of phosphors with application in solid-state lighting, displays, lasers, bioimaging, and sensors.^1−3^ One of the first Mn^4+^-doped phosphors studied back in the early 1960s was the Mn^4+^-doped corundum (sapphire), α-Al_2_O_3_:Mn^4+^,^4^ which has been considered as an alternative of a first laser material ruby (α-Al_2_O_3_:Cr^3+^). Following this, many oxide compounds activated with Mn^4+^ ions were investigated.^2^ The spectroscopic properties of transition-metal ions, like Mn^4+^ or Cr^3+^ (both 3d^3^ configuration), are strongly influenced by the surrounding crystal field. Emission of Mn^4+^ ions, which usually have octahedral coordination, exhibits a sharp line due to the spin-forbidden ^2^E → ^4^A_2_ transition. This is due to the strong crystal field experienced typically by the Mn^4+^ ions because of the highly effective positive charge of the cation. The broadband emission caused by the spin-allowed ^4^T_2_ → ^4^A_2_ transition usually is not observed for Mn^4+^, unlike Cr^3+^.

Ga_2_O_3_–Al_2_O_3_ alloys (solid solutions) represent an interesting host material because their crystal structure and physical properties can be changed by modifying the composition in a wide range.^5^ A recently published report on the tuning of the photoluminescence properties of Cr^3+^ ions in the (Ga_1–*x*_Al_*x*_)_2_O_3_ host lattice gives information on the effect of Al for Ga substitution.^6^ In particular, it was shown that the replacement of Al by Ga in this host lattice weakens the crystal-field strength experienced by Cr^3+^ ions, so that the broadband emission due to the ^4^T_2_ → ^4^A_2_ transition in Cr^3+^ occurs at room temperature alongside the ^2^E → ^4^A_2_ transition. The photoluminescence properties of Cr^3+^ ions in β-Ga_2_O_3_ are also well-documented, and the β-Ga_2_O_3_:Cr^3+^ phosphor has been proposed as a promising high-pressure calibrant for a diamond anvil cell (DAC),^7^ for near-IR-tunable laser applications,^8,9^ for artificial lighting for agriculture,^10^ and for noncontact luminescence thermometry.^11−13^ The opposite effect, i.e., strengthening of the crystal field, can be achieved by applying a hydrostatic pressure, which has been demonstrated in a number of Cr^3+^-doped compounds.^6,14−16^

To the best of our knowledge, up to now, there have been no studies of Mn^4+^ emission in Ga_2_O_3_–Al_2_O_3_ alloys, except for pure α-Al_2_O_3_. Therefore, it is of interest to explore what may happen with the emission of Mn^4+^ when the α-Al_2_O_3_ crystal lattice is modified by the addition of Ga. At such a modification, one can expect the influence of at least three factors. The first is a weakening of the crystal-field strength experienced by Mn^4+^ ions in the corundum structure modified by gallium. The second is a decrease of the band gap of the host lattice, which will occur as the Ga content increases.^5^ Finally, the corundum structure of the host should switch to the monoclinic β-Ga_2_O_3_-type structure at about 20% Ga.^5,6^ Because of the increase of the Al(Ga)–O distances, structure alteration can cause a weakening of the crystal-field strength to the extent that the ^4^T_2_ → ^4^A_2_ emission may become possible even for Mn^4+^ ions. It would be also interesting to verify the degree of covalency for Mn^4+^–O^2–^ bonding in Ga_2_O_3_–Al_2_O_3_ alloys using as a criterion the position of the spin-forbidden ^2^E → ^4^A_2_ emission line.^17,18^

Aiming to gain insight into these issues, we synthesized a series of original microcrystalline phosphor materials with nominal composition (Al_1–*x*_Ga_*x*_)_2_O_3_:Mn(0.05 atom %),Mg (0.05 atom %) with *x* = 0, ..., 1.0 and investigated their crystal structure and the photoluminescence properties of Mn^4+^ ions in the temperature range from 4.4 to 500 K. The studies are complemented by the measurements of the luminescence properties of β-Ga_2_O_3_:Mn^4+^ at hydrostatic pressures of up to 30 GPa.

## Experimental Methods

A series of Mn- and Mg-codoped aluminum–gallium oxides of nominal composition (Al_1–*x*_Ga_*x*_)_2_O_3_:Mn(0.05 atom %),Mg(0.05 atom %) with *x* = 0, 0.05, 0.1, 0.15, 0.2, 0.25, 0.50. 0.75, and 1.0 were produced by a sol–gel citrate method from Al(NO_3_)_3_·9H_2_O, Mn(NO_3_)_2_·4H_2_O, and Mg(NO_3_)_2_·6H_2_O as the initial reagents. The metallic Ga dissolved in HNO_3_ was used as a Ga source. Appropriate aliquots of metal nitrate solutions corresponding to a nominal composition of a sample were mixed in a magnetic stirrer for 30 min. After that, a water solution of citric acid was added to the reaction mixture, ensuring that the molar ratio of metals to citric acid was equal to 1:2. The prepared solutions were evaporated at a temperature of 353 K and dried at 373 K to form a polymer gel. Heat treatment of the obtained product was carried out in several stages: at temperatures of 573 and 725 K for 30 min to remove the organic component and subsequently at 973, 1173, and 1473 K for 4–7 h to ensure crystallization and formation of the desired phase. A final heat treatment of the product was performed at 1773 K for 7 h.

X-ray diffraction (XRD) characterization of the synthesized materials was performed with an Aeris benchtop powder diffractometer (Malvern Panalytical) equipped with a PIXcel^1D^ strip detector. Some measurements were made using a high-resolution X’Pert MRD diffractometer (Philips). Experimental diffraction data were collected using filtered Cu Kα radiation (λ = 1.54185 Å) or a monochromated Cu Kα_1_ beam (λ = 1.54056 Å) in a 2θ range of 10–105° with a 2θ step of 0.01°. Crystal structure parameters (unit cell dimensions, coordinates, and displacement parameters of atoms) were derived from the experimental XRD patterns by full profile Rietveld refinement using the *WinCSD* software package.^19^

The photoluminescence (PL) and photoluminescence excitation (PLE) spectra were measured using a Horiba/Jobin-Yvon Fluorolog-3 spectrofluorometer with a 450 W continuous-wave xenon lamp for excitation. The emission was detected by a Hamamatsu R928P photomultiplier operating in a photon-counting mode. The PL spectra were corrected for the spectral response of the used system. The luminescence decay kinetics were measured using the same Fluorolog-3 spectrofluorometer, with the excitation light modulated by a mechanical chopper. Spectroscopic measurements in the temperature range 4.4–330 K were carried out on a Janis continuous-flow liquid-helium cryostat using a Lake Shore 331 temperature controller. Studies in the range 295–500 K were done in air using a compact resistive heater and an Eurotherm 902 temperature controller. The photoluminescence quantum efficiency (QE) was estimated as the ratio of the number of emitted photons to that of absorbed photons, similar to that described in ref (20).

The high-pressure luminescence measurements were carried out on a miniature DAC from easyLab placed in a CF 200 Oxford Instruments continuous-flow cryostat with an ITC4 Oxford Instruments temperature controller. The studied powder sample was ground in a mortar before loading into the cell in order to have a finer powder. Because of the fact that the studied β-Ga_2_O_3_:Mn sample contained Cr^3+^ ions as unintentional dopant, it was decided not to introduce a ruby as a pressure sensor but to use the R_1_-line position of Cr^3+^ in β-Ga_2_O_3_ for pressure calibration, as reported in ref (7). A methanol–ethanol mixture in a volume ratio of 5:1 was used as the pressure-transmitting medium. The luminescence was collected in a backscattering geometry using a Yobin Yvon-Spex Triax 320 monochromator equipped with a Spectrum One liquid-nitrogen-cooled CCD camera. In this experiment, the luminescence was excited by a 405 nm emission from 100 mW diode laser or by a 325 nm line from a 20 mW He–Cd laser.

High-energy-resolution fluorescence-detected X-ray absorption near-edge structure (HERFD-XANES) spectra were collected using a 1m X-ray emission Johann spectrometer at the Diamond Light Source on the I20 scanning beamline.^21^ For this experiment, three Ge(333) analyzer crystals were used to monitor the intensity of the Mn Ka emission line as a function of the incident energy. The spectrometer and monochromator were calibrated by measuring the Ka-line (5898.8 eV) and K-edge (6539 eV), respectively, from a Mn foil, and an elastic measurement confirmed that energy resolution of the measurements overcomes the core–hole lifetime broadening.

## Results and Discussion

### Phase Composition and Crystal Structure Parameters

**Figure 1:**
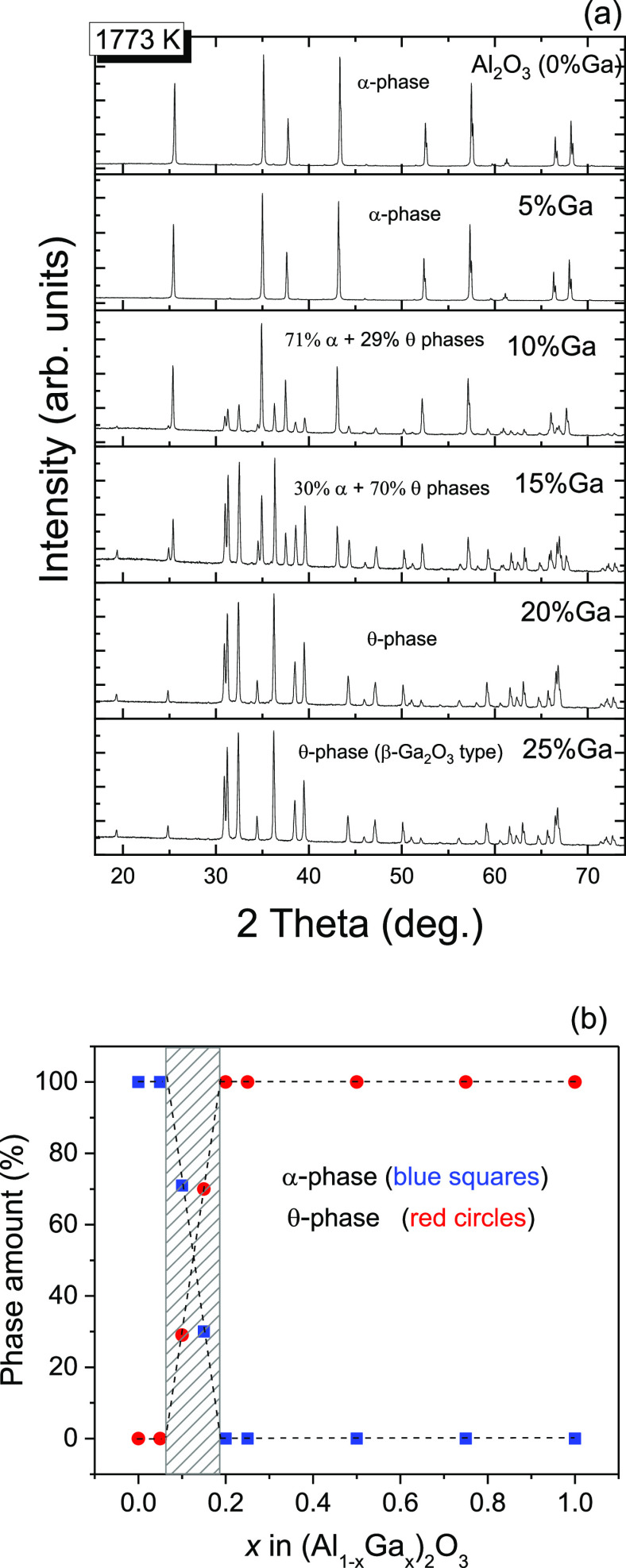
XRD patterns (a) and phase compositions of the (Al_1–*x*_Ga_*x*_)_2_O_3_:Mn,Mg powders (b) illustrating phase evolution in the (Al_1–*x*_Ga_*x*_)_2_O_3_ series versus Ga content. The phase composition for 10 and 15% Ga samples was derived by quantitative full profile Rietveld analysis.

Examination of the XRD patterns for a series of (Al_1–*x*_Ga_*x*_)_2_O_3_:Mn,Mg materials heat-treated at 1473 K reveled the formation of a corundum-type α-Al_2_O_3_ structure as the main phase for the Al-rich specimens with *x* ≤ 0.15 and a pure monoclinic θ-Al_2_O_3_ (β-Ga_2_O_3_) phase for the Ga-rich samples with *x* ≥ 0.5. For the intermediate compositions with *x* = 0.02 and 0.25, a metastable δ-Al_2_O_3_ polymorph was identified as the main phase. The phase relationships in the (Al_1–*x*_Ga_*x*_)_2_O_3_ system changed considerably after the final heat treatment of the samples at 1773 K for 7 h. Pure corundum and β-Ga_2_O_3_-type structures were found in the samples with *x* ≤ 0.05 and *x* ≥ 0.2, respectively, whereas the samples with intermediate compositions (*x* = 0.10 and 0.15) consisted of a mixture of these two phases in different proportions (Figure 1a). Thus, it was confirmed that, in the (Al_1–*x*_Ga_*x*_)_2_O_3_ system, the morphotropic phase transition between the rhombohedral corundum and monoclinic β-Ga_2_O_3_ types of structures occurs at 0.05 < *x* < 0.20 (Figure 1b). These materials were taken for further structural and luminescent investigations.

**Figure 2:**
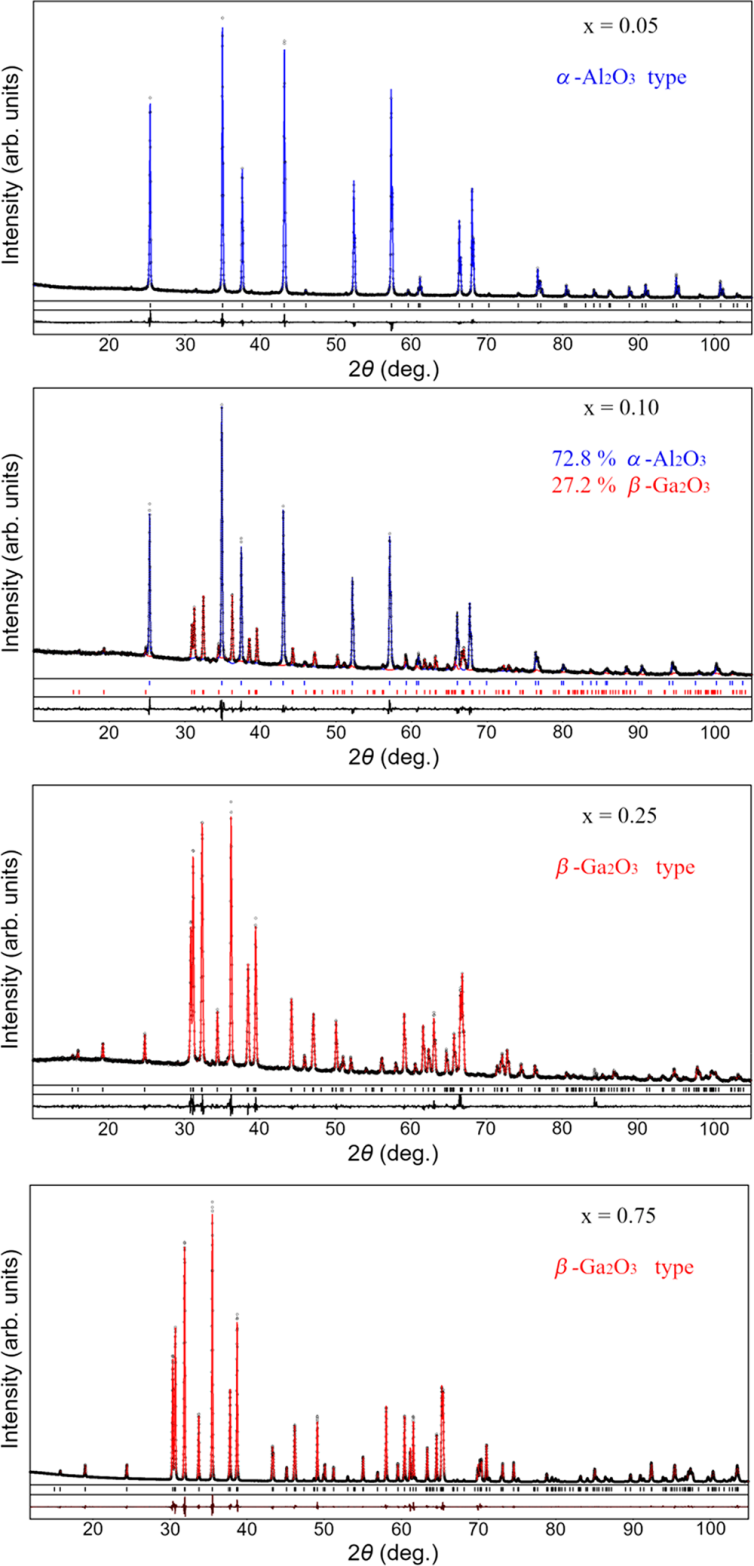
Graphical results of Rietveld refinement of (Al_1–*x*_Ga_*x*_)_2_O_3_:Mn,Mg structures with *x* = 0.05, 0.10, 0.25, and 0.75 (from top to bottom). The experimental XRD patterns (small black circles) are shown in comparison with the calculated patterns for the α-Al_2_O_3_ and β-Ga_2_O_3_ types of structures (blue and red lines, respectively).

The phase compositions and crystal structures of the investigated materials were confirmed by full profile Rietveld refinement, performed in space groups *R*3*c* and *C*2/*m* for the corresponding samples. In all cases, an excellent agreement between the experimental and calculated XRD profiles was achieved (see the examples in Figure 2). The refined structural parameters for selected representatives of the α-Al_2_O_3_ and β-Ga_2_O_3_ types of structures in the (Al_1–*x*_Ga_*x*_)_2_O_3_:Mn,Mg series are summarized in Table 1.

**Table 1:** Lattice Parameters, Coordinates, and Displacement Parameters of Atoms in the Rhombohedral and Monoclinic Structures of Selected (Al_1–*x*_Ga_*x*_)_2_O_3_:Mn,Mg (*x* = 0.05, 0.10, 0.25, and 0.75) Samples

lattice param	atoms, sites	*x*/*a*	*y*/*b*	*z*/*c*	*B*_iso/eq_, Å^2^	occupancy
(Al_0.95_Ga_0.05_)_2_O_3_:Mn,Mg (*R*3*c*); *R*_I_ = 0.026, *R*_P_ = 0.049						
*a* = 4.76550(6) Å	Al, 12c	0	0	0.35218(8)	1.79(3)	0.953(4) Al^3+^ + 0.047(4) Ga^3+^
*c* = 13.0062(2) Å	O, 18e	0.3049(4)	0	^1^/_4_	1.79(5)	O^2–^
(Al_0.90_Ga_0.10_)_2_O_3_:Mn,Mg (*R*3*c*,72.8 wt.%); *R*_I_ = 0.034, *R*_P_ = 0.111						
*a* = 4.78804(9) Å	Al, 12c	0	0	0.35296(7)	1.36(3)	0.895(2) Al^3+^0.105(2) Ga^3+^
*c* = 13.0511(3) Å	O, 18e	0.3073(4)	0	^1^/_4_	2.02(5)	O^2–^
(Al_0.90_Ga_0.10_)_2_O_3_:Mn,Mg (*C*2/*m*, 27.2 wt %); *R*_I_ = 0.063, *R*_P_ = 0.111						
*a* = 11.8816(3) Å	Al1, 4i	0.0912(4)	0	0.8015(10)	0.59(9)	0.890(7) Al^3+^ + 0.110(7) Ga^3+^
*b* = 2.94055(9) Å	Al2, 4i	0.3412(3)	0	0.6826(9)	1.51(13)	0.897(8) Al^3+^ + 0.103(8) Ga^3+^
*c* = 5.6685(2) Å	O1, 4i	0.1604(7)	0	0.102(2)	2.4(2)	O^2–^
β = 104.080(2)°	O2, 4i	0.4913(6)	0	0.2517(14)	3.2(3)	O^2–^
	O3, 4i	0.8256(6)	0	0.414(2)	2.7(2)	O^2–^
(Al_0.75_Ga_0.25_)_2_O_3_:Mn,Mg (*C*2/*m*); *R*_I_ = 0.029, *R*_P_ = 0.078						
*a* = 11.9040(2) Å	Al1, 4i	0.0911(2)	0	0.7957(3)	1.45(3)	0.641(9) Al^3+^ + 0.359(9) Ga^3+^
*b* = 2.94661(4) Å	Al2, 4i	0.3423(1)	0	0.6838(3)	1.24(4)	0.842(8) Al^3+^ + 0.158(8) Ga^3+^
*c* = 5.67849(6) Å	O1, 4i	0.1593(3)	0	0.1106(7)	2.23(9)	O^2–^
β = 104.080(1)°	O2, 4i	0.4952(3)	0	0.2568(5)	2.39(10)	O^2–^
	O3, 4i	0.8280(3)	0	0.4351(7)	1.86(8)	O^2–^
(Al_0.25_Ga_0.75_)_2_O_3_:Mn,Mg (*C*2/*m*); *R*_I_ = 0.016, *R*_P_ = 0.056						
*a* = 12.11953(6) Å	Al1, 4i	0.09053(5)	0	0.7947(1)	0.72(1)	0.18(1) Al^3+^ + 0.82(1) Ga^3+^
*b* = 3.01145(2) Å	Al2, 4i	0.34162(5)	0	0.6862(1)	0.70(2)	0.32(1) Al^3+^ + 0.68(1) Ga^3+^
*c* = 5.77046(3) Å	O1, 4i	0.1632(2)	0	0.1064(5)	1.20(5)	O^2–^
β = 103.963(1)°	O2, 4i	0.4962(2)	0	0.2560(4)	1.23(6)	O^2–^
	O3, 4i	0.8284(2)	0	0.4353(4)	0.95(5)	O^2–^

**Figure 3:**
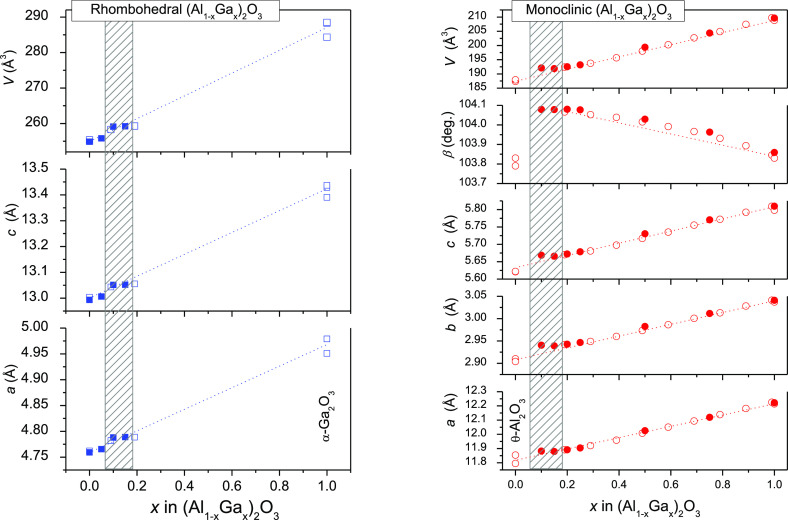
Concentration dependence of the lattice parameters and unit cell volumes of the rhombohedral (left) and monoclinic (right) phases in the (Al_1–*x*_Ga_*x*_)_2_O_3_:Mn,Mg series (solid symbols) in comparison with the literature data for the metastable α-Ga_2_O_3_, θ-Al_2_O_3_^22^ and Cr^3+^-doped (Al_1–*x*_Ga_*x*_)_2_O_3_^6^ (open symbols). Hatched areas indicate a two-phase region of the system.

The lattice parameters and unit cell volumes of rhombohedral and monoclinic modifications of the (Al_1–*x*_Ga_*x*_)_2_O_3_:Mn,Mg structure increase systematically with increasing Ga content in the corresponding series (Figure 3). The structural parameters of the Mn/Mg-doped (Al_1–*x*_Ga_*x*_)_2_O_3_ solid solution studied agree well with the reference data for the end members of the system, i.e., α-Al_2_O_3_ and β-Ga_2_O_3_ and their metastable polymorphs α-Ga_2_O_3_ and θ-Al_2_O_3_, which are collected in Pearson’s crystal structure database,^22^ as well as with the recently reported data for Cr^3+^-doped (Al_1–*x*_Ga_*x*_)_2_O_3_^6^ (see the left and right panes of Figure 3, respectively).

**Figure 4:**
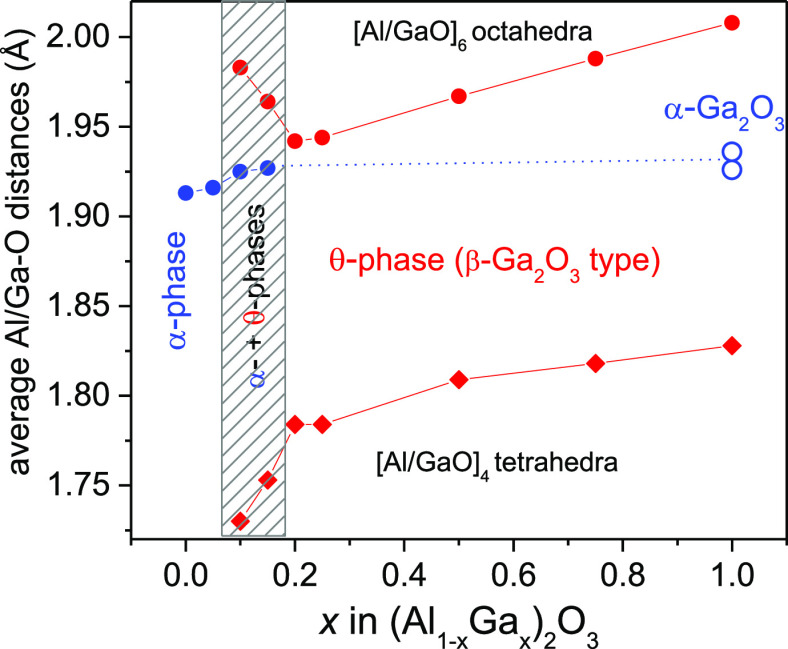
Average interatomic distances inside (Al/Ga)O_6_ octahedra and (Al/Ga)O_4_ tetrahedra in rhombohedral and monoclinic (Al_1–*x*_Ga_*x*_)_2_O_3_:Mn,Mg structures. The corresponding intraoctahedral distances in the metastable α-Ga_2_O_3_ structure^22^ are shown as open blue circles for comparison. The hatched area indicates a two-phase region of the system.

The average interatomic distances inside (Al/Ga)O_6_ octahedra and (Al/Ga)O_4_ tetrahedra in rhombohedral and monoclinic (Al_1–*x*_Ga_*x*_)_2_O_3_ are shown in Figure 4. As can be seen from the figure, the interatomic distances inside (Al/Ga)O_6_ octahedra and (Al/Ga)O_4_ tetrahedra also increase in the (Al_1–*x*_Ga_*x*_)_2_O_3_:Mn,Mg series, in both the rhombohedral and monoclinic structures. Note that the average intraoctahetral Ga–O distances in the monoclinic β-Ga_2_O_3_ structure are considerably larger than those in the metastable corundum-type α-Ga_2_O_3_ structure.

### PL and PLE of Mn^4+^ in Ga_2_O_3_–Al_2_O_3_ Alloys

**Figure 5:**
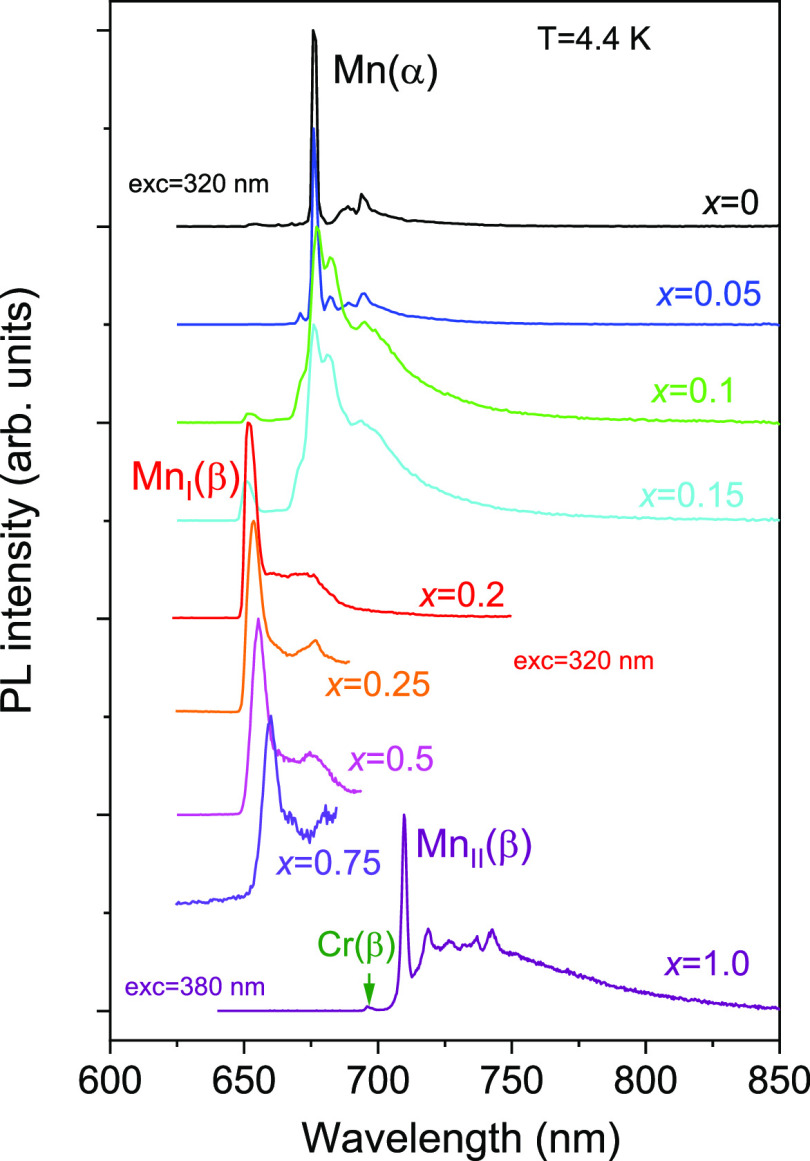
Normalized low-temperature PL spectra of Mn^4+^ ions in the (Al_1–*x*_Ga_*x*_)_2_O_3_ solid solutions (*x* = 0, ..., 1.0) calcined at 1773 K.

Figure 5 demonstrates the low-temperature Mn^4+^ emission spectra observed for different compositions of (Al_1–*x*_Ga_*x*_)_2_O_3_:Mn,Mg. Inspection of the spectra reveals three different types of Mn^4+^ centers in the studied materials depending on their chemical composition. The first one, marked as Mn(α), corresponds to the Mn^4+^ ions in the corundum-type structure of the host lattice. A zero-phonon-line (ZPL) emission of this center for *x* = 0 peaks at 676.3 nm, which coincides with the known Mn^4+^ emission in α-Al_2_O_3_.^4,23^ This type of Mn^4+^ center is observed in the studied compounds, where with a corundum phase exists (*x* = 0, ..., 0.15; see the previous chapter). For the *x* = 0.1 and 0.15 compositions, besides Mn(α), another type of Mn^4+^ center, denoted as Mn_I_(β), also starts to appear. The sole emission of the Mn_I_(β) center is observed in the single-phase monoclinic compounds with *x* = 0.2, ..., 0.75. Admittedly, the Mn_I_(β) center is generically associated with the monoclinic-type structure of the (Al_1–*x*_Ga_*x*_)_2_O_3_ host lattice. The ZPL emission peak for Mn_I_(β) slightly shifts from 651.0 to 659.9 nm as the host composition is changed from *x* = 0.15 to 0.75. Judging from the position of the ZPL of Mn_I_(β) with respect to Mn(α), one can conclude that the Mn–O bonds in monoclinic-type solid solutions have significantly lower covalency in comparison with the corundum-type (Al_1–*x*_Ga_*x*_)_2_O_3_. At the same time, the covalency of the Mn–ligand bonds in the monoclinic host lattice slightly increases as the Ga content increases. It should also be noted that the emission efficiency of the Mn_I_(β) center is much lower than that of Mn(α), and it gradually decreases as the Ga content increases (Table 2). Therefore, the emission intensity of Mn_I_(β) in the double-phase samples with *x* = 0.1 and 0.15 is much less than the Mn(α) intensity despite the fact that the amounts of the monoclinic phase in these samples are 27% and 70%, respectively.

**Table 2:** Internal Quantum Efficiency of the (Al_1–*x*_Ga_*x*_)_2_O_3_:Mn^4+^ Samples (Calcined at 1773 K) Measured at 320 nm Excitation at Room Temperature

sample composition (*x* value)	QE, %
0	22.6 ± 1.0
0.05	20.6 ± 0.5
0.1	16.1 ± 1.0
0.15	18.2 ± 1.0
≥0.2	low, not measured

Interestingly, a completely different Mn^4+^ center (λ_ZPL_ = 709.7 nm at *T* = 4.4 K), marked as Mn_II_(β), was found in β-Ga_2_O_3_ (*x* = 1.0), which has the same type of monoclinic structure. It should be stressed that no emission of the Mn_II_(β) center was observed in any of the solid solutions with *x* from 0 to 0.75. At the same time, no traces of the Mn_I_(β) center emission were found in β-Ga_2_O_3_ (*x* = 1.0). The fact that the Mn_II_(β) center is revealed only in pure β-Ga_2_O_3_ indicates that this center may represent Mn^4+^ ions occupying Ga sites. In contrast, in monoclinic β-(Al_1–*x*_Ga_*x*_)_2_O_3_, the emission of the Mn_I_(β) center is observed over a broad range of *x*, indicating that the nearest environment of Mn^4+^ is only mildly affected by Ga for Al substitution. We anticipate that the random presence of smaller Al^3+^ ions in a second coordination sphere allows one to accommodate the distortion in the nearest environment of Mn^4+^ caused by the change of the Mn–ligand distances. This assumption is based on the similarity of the ionic radii of the Mn^4+^ and Al^3+^ ions. The question remains whether the octahedral or tetrahedral Ga site is occupied by Mn^4+^ ions in pure β-Ga_2_O_3_.

**Figure 6:**
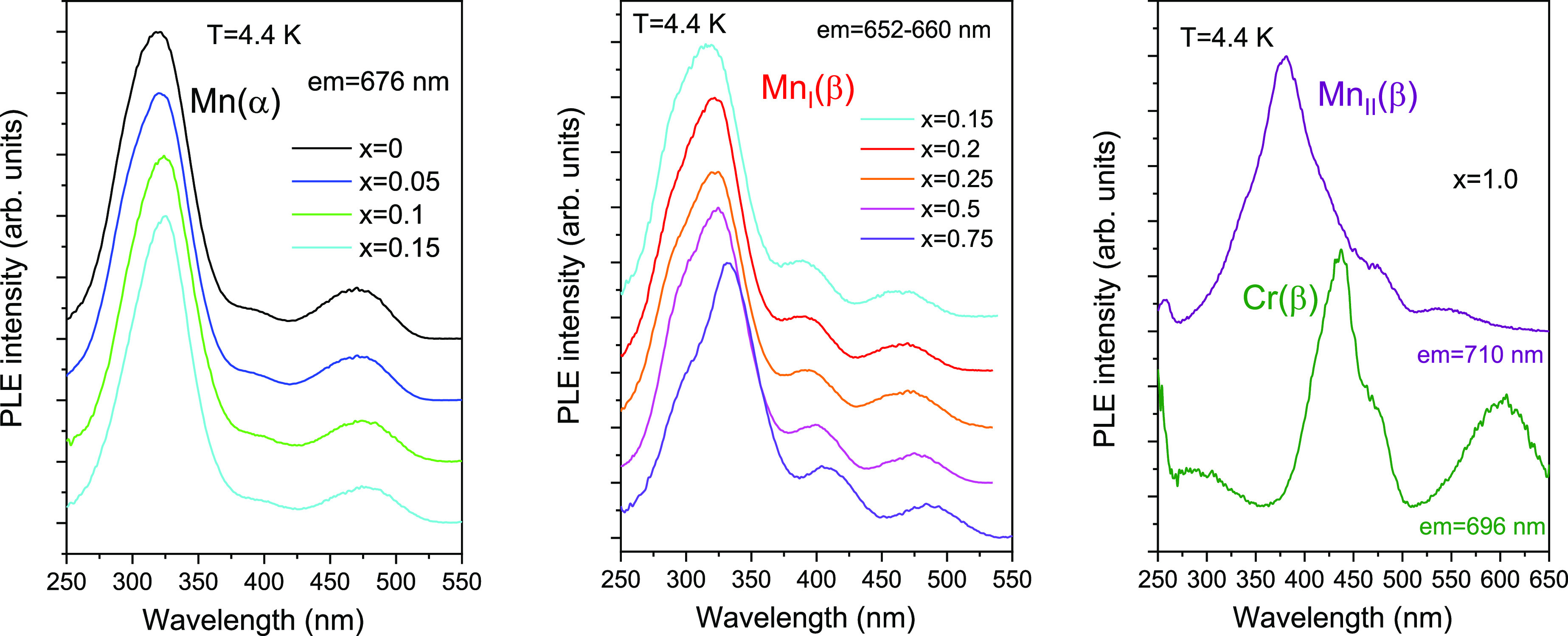
Characteristic low-temperature PLE spectra of the studied (Al_1–*x*_Ga_*x*_)_2_O_3_:Mn solid solutions calcined at 1773 K.

**Figure 7:**
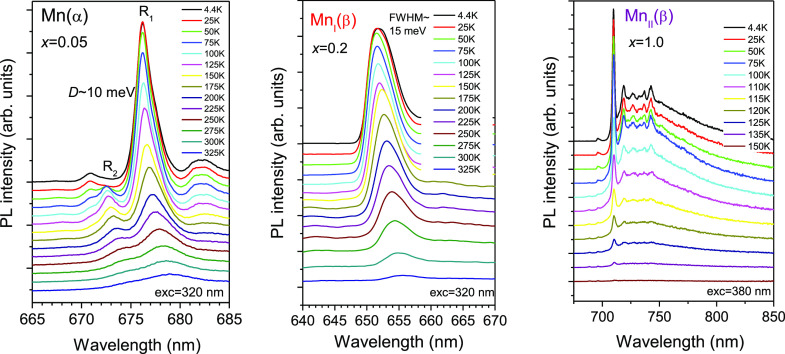
Temperature dependence of the PL spectra of different Mn^4+^ centers measured for (Al_1–*x*_Ga_*x*_)_2_O_3_:Mn with *x* = 0.05, 0.2, and 1.0.

Characteristic PLE spectra of the observed Mn(α), Mn_I_(β), and Mn_II_(β) centers are shown in Figure 6. The plots in the figure show that, within each structural type (α and β), the charge-transfer (CT) (∼320 nm) and ^4^A_2_ → ^4^T_2_ (∼470 nm) excitation bands slightly shift toward lower energies with increasing Ga content. This means that the crystal-field strength for Mn^4+^ ions decreases as the Ga content rises in both the corundum and monoclinic types of structures, which is expected in view of the increasing average interatomic Al(Ga)–O distances in the octahedra (Figure 4). The excitation spectrum of the Mn_II_(β) center in β-Ga_2_O_3_ stands alone from the mixed compositions. Here the strong excitation band (most probably the O–Mn^4+^ CT band) is significantly red-shifted, exhibiting a peak at about 380 nm, while the lowest-energy excitation band centered at 540 nm is likely due to ^4^A_2_ → ^4^T_2_ transitions. It should be noted that, because of the relatively low emission intensity of the Mn_I_(β) and Mn_II_(β) centers, the luminescence of the unintentional Cr^3+^ impurity also becomes visible in the compounds with higher Ga content. In particular, Cr^3+^ in the studied β-Ga_2_O_3_ (*x* = 1.0) sample is manifested through the ^4^A_2_ → ^4^T_1_ (∼435 nm) and ^4^A_2_ → ^4^T_2_ (∼600 nm) excitation bands (Figure 6), as well as the ZPL (^2^E → ^4^A_2_) emission at ∼696 nm (Figure 5).^11,12^ As can be seen in Figure 7, the ZPL of the Mn(α) center exhibits splitting (R_1_ and R_2_ lines) of ca. 10 meV. For the Mn_I_(β) center, no splitting can be seen in the luminescence spectra because of a larger width of the ZPL (fwhm ∼15 meV). Nonetheless, it is not unreasonable to assume a few millielectronvolts splitting of this state because of distortion of the local environment of Mn^4+^. This splitting is essential to explain the temperature changes of the decay time constant (see below), and, therefore, we anticipated that it is equal to 2 meV. The ZPL of the Mn_II_(β) center is narrower (fwhm ∼6 meV) without any hint of splitting.

The Mn_II_(β) emission needs to be considered in more detail. The PL spectrum of this emission consists of a sharp line with some vibrionic sidebands superimposed on a broad band stretching of up to 850 nm (Figure 5), allowing one to assume that Mn^4+^ ions here are in a significantly weaker (intermediate) crystal field, so that a superposition of the emissions from the ^2^E and ^4^T_2_ states can occur. As can be seen from Figure 7, the intensity of the broad band around 740 nm with respect to the sharp line at 709 nm increases as the temperature increases even though the overall emission intensity is quickly quenched at *T* ≥ 100 K. Such a temperature evolution of the PL spectrum shape is inherent in the case of superposition of the emissions from the ^2^E and ^4^T_2_ states; however, it is also possible for the phonon-assisted ^2^E → ^4^A_2_ emission only.^2,3^

It is unusual that the ZPL emission of Mn_II_(β) (709.7 nm at *T* = 4.4 K) is red-shifted with respect to the Cr^3+^ ZPL (∼696 nm) in the same β-Ga_2_O_3_ (*x* = 1.0) host. This can be explained assuming that the Mn_II_(β) center corresponds to Mn^4+^ ions in the tetrahedral Ga sites of β-Ga_2_O_3_, unlike Cr^3+^ occupying octahedral sites in β-Ga_2_O_3_. It should be noted that the Mn^4+^ ions in tetrahedral coordination were already reported in the natural mineral cordierite.^24^ A higher covalency of the Mn_II_(β) center, even higher than that of the octahedral Mn(α) or Cr(β) centers, can also favor the tetrahedrally coordinated Mn^4+^ ions in β-Ga_2_O_3_. If we assume that the Mn_II_(β) center corresponds to Mn^4+^ ions in the tetrahedral Ga sites of β-Ga_2_O_3_, the ZPL emission at 709.7 nm should be caused by the spin-forbidden ^2^E → ^4^T_1_ transition, which corresponds to the 3d^7^ electron configuration in the octahedral crystal field.

### Pressure-Dependent PL of Mn^4+^ in β-Ga_2_O_3_

**Figure 8:**
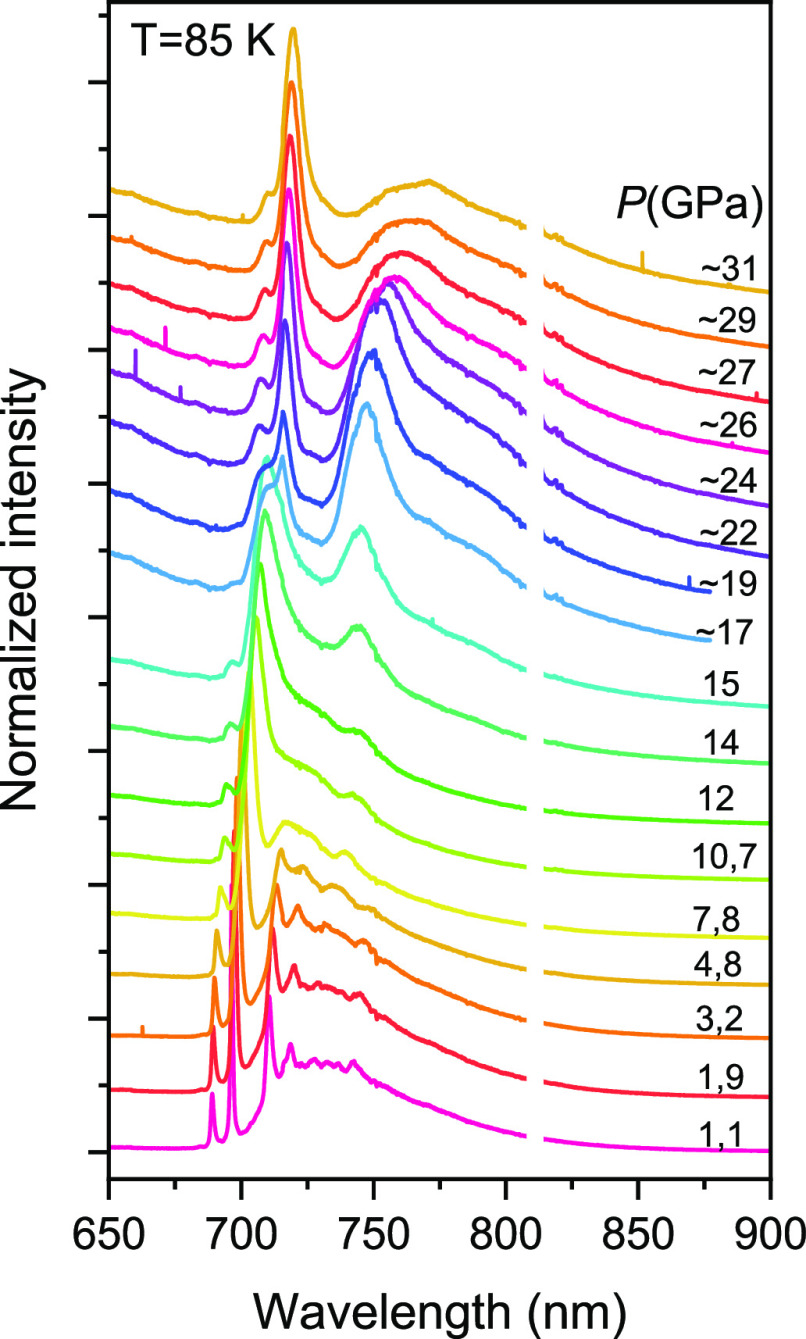
Pressure dependence of the PL spectra of β-Ga_2_O_3_:Mn measured at 405 nm excitation at *T* = 85 K.

To clarify the nature of the Mn_II_(β) center in β-Ga_2_O_3_, we measured the pressure-dependent PL spectra for this sample. The results of this experiment are shown in Figure 8. Here we used 405 nm excitation to strengthen the R lines from Cr^3+^ observed at 695.6 and 688.9 nm at *T* = 85 K and ambient pressure, which was used for pressure monitoring. As one can see from the figure, the Mn-related sharp line at about 709 nm decreases in intensity relative to the broadband emission around 740 nm and merges into one structureless band as the pressure increases. This testifies unambiguously that the Mn_II_(β) center emission in β-Ga_2_O_3_ is caused by the spin-forbidden transition from the ^2^E level, whereas the broad band around 740 nm with some fine structure at ambient pressure is caused by phonon-assisted components of the same transition. It should be mentioned that a similar shape of the Mn^4+^ emission spectrum with a narrow ZPL peak and a broad band with weakly expressed thin structure from the Stokes side of ZPL has been also observed in Sr_4_Al_14_O_25_:Mn^4+^.^25^

As can be seen in Figure 8, at pressures starting at about 10 GPa, new Cr- and Mn-related lines appear simultaneously with the disappearance of the corresponding lines in β-Ga_2_O_3_. This is related evidently with the pressure-induced phase transformation of the monoclinic β-Ga_2_O_3_ to the rhombohedral α-Ga_2_O_3_.^26,27^ The transformation is almost completed at pressures of about 19 GPa, which is in agreement also with the results reported for Ga_2_O_3_:Cr.^6^ After pressure release, the spectrum shape does not return to the previous one for β-Ga_2_O_3_, which agrees with the irreversible pressure-induced transformation from the monoclinic to rhombohedral phase.^26^ Consequently, the application of hydrostatic pressure in a DAC of ≥19 GPa allowed us to obtain the corundum-like α-Ga_2_O_3_:Mn, which can be synthesized only at high pressures.^28^ It is of interest to note that alloying of β-Ga_2_O_3_ with Al exerts a chemical pressure that decreases the external physical pressure required for the b®a structure transformation, so when the Al content exceeds 80%, ambient pressure is sufficient to obtain the high-density corundum-like structure.

**Figure 9:**
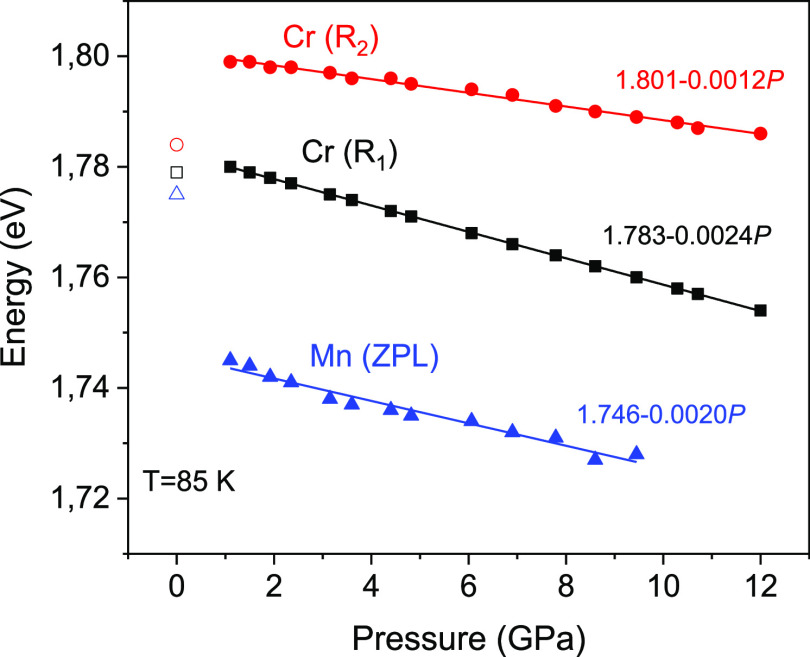
Shift of the ZPL position with hydrostatic pressure for the Mn^4+^ and Cr^3+^ centers in β-Ga_2_O_3_ measured at *T* = 85 K (solid symbols) with the corresponding linear approximation (solid lines) in eV and GPa units. Open symbols represent the corresponding ZPL positions in α-Ga_2_O_3_ at ambient pressure.

**Figure 10:**
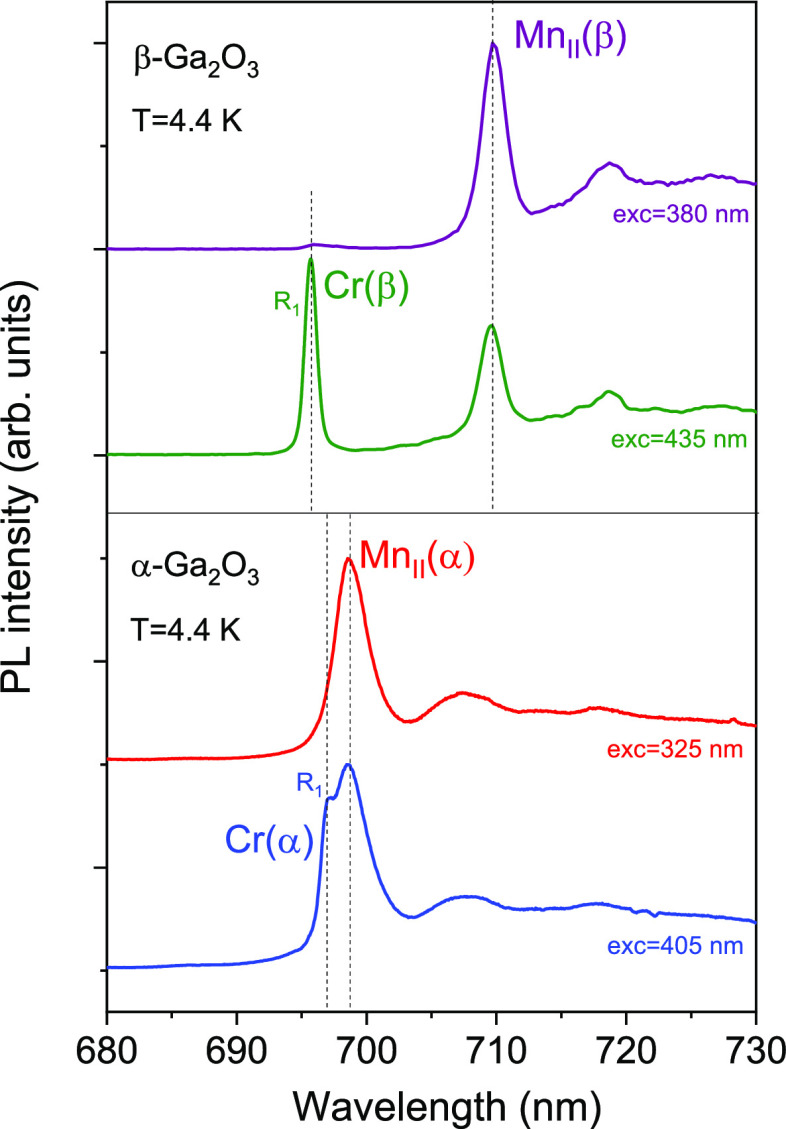
ZPL emission of Mn^4+^ and Cr^3+^ ions in β-Ga_2_O_3_ and α-Ga_2_O_3_ at *T* = 4.4 K at ambient pressure.

The pressure-induced shift of the ZPL position for the Mn^4+^ and Cr^3+^ centers in β-Ga_2_O_3_ is shown in Figure 9. The corresponding pressure coefficients are summarized in Table 3 in comparison with other Mn^4+^-doped oxide materials. As one can see from the table, the pressure coefficient for Mn^4+^ in β-Ga_2_O_3_ does not exceed those for other materials, where Mn^4+^ ions are in octahedral coordination, which rules out possible tetrahedral coordination of the Mn^4+^ centers in β-Ga_2_O_3_. As a reminder, in the case of tetrahedral coordination of the Mn^4+^ centers (which corresponds to the 3d^7^ electron configuration in the octahedral crystal field), the emitting ^2^E level should move down much more rapidly as the crystal field (pressure) increases. The characteristic ZPL emission spectra of the Mn^4+^ and Cr^3+^ centers in the pressure-created α-Ga_2_O_3_ in comparison with the starting β-Ga_2_O_3_ are shown in Figure 10. Here and after the Mn^4+^ center in α-Ga_2_O_3_ is marked as Mn_II_(α). It should be mentioned that we were unable to determine exactly the pressure coefficient for Mn^4+^ in α-Ga_2_O_3_ because at pressures above 15 GPa the R lines of Cr^3+^ in β-Ga_2_O_3_ used for pressure calibration disappeared almost completely (Figure 9). Therefore, the pressure values after the phase transition (*P* ≥ 15 GPa) were estimated only roughly based on the external force applied to the diamonds.

**Table 3:** Pressure Coefficients of the Mn^4+^ and Cr^3+^ ZPL (^2^E → ^4^A_2_) Energies in Some Oxide Phosphors

material	activator	Δ*E*/Δ*P*, meV/GPa	*T*, K	ref
α-Al_2_O_3_	Mn^4+^	–1.05 (R_1_)	300	(29)
		–1.06 (R_2_)		
YAlO_3_ (YAP)		–1.43	10	(30)
Gd_3_Ga_5_O_12_ (GGG)		–2.32 (site 1)	15	(31)
		–1.85 (site 2)		
Sr_4_Al_14_O_25_		–2.64	300	(25)
Mg_2_TiO_4_		–2.4	50	(32)
β-Ga_2_O_3_		–2.0	85	this work
α-Ga_2_O_3_		not estimated	85	this work
β-Ga_2_O_3_	Cr^3+^	–2.4 (R_1_)	85	(7)
		–0.6 (R_2_)		
		–2.4 (R_1_)	85	this work
		–1.2 (R_2_)		
β-Ga_1.58_Al_0.4_O_3_		–1.5 (R_1_)	300	(5)
		–1.0 (R_2_)		
β-Ga_0.38_Al_1.6_O_3_		–1.28 (R_1_)	300	(6)
		–1.1 (R_2_)		
α-Al_2_O_3_		–0.9 (R_1_)	300	(33)
		–1.0 (R_2_)		
		–0.9 (R_1_)	300	(34)
α-Ga_1.58_Al_0.4_O_3_		–1.09	300	(6)
α-Ga_0.38_Al_1.6_O_3_		–1.13	300	(6)

**Figure 11:**
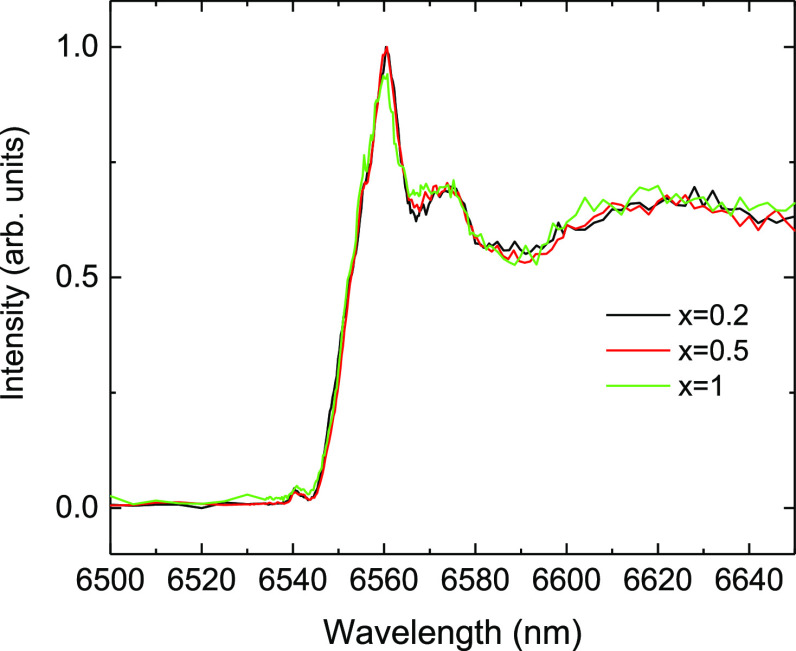
HERFD-XANES spectra of (Al_1–*x*_Ga_*x*_)_2_O_3_:Mn,Mg (*x* = 0.2, 0.5 and 1) at the Mn K-edge.

Aiming to get an additional clue regarding the surroundings of the Mn^4+^ centers in β-Ga_2_O_3_, we turned our attention to X-ray absorption spectroscopy, which can be used as a sensitive local probe of the electronic structure of transition-metal ions.^35^ The HERFD-XANES spectra were measured from the solid solutions (Al_1–*x*_Ga_*x*_)_2_O_3_:Mn,Mg (*x* = 0.2–1), which exhibit the same monoclinic crystal structure, by monitoring the intensity of the Kα-line across the K-edge (1s→) of Mn. The measured spectra are displayed in Figure 11. The spectra show a preedge peak at 6540 eV due to the promotion of 1s electron to 3d orbital. The edge and near-edge structures are due to transitions of the core electron (1s) to higher unoccupied states and continuum of the conduction band. A comparison of the spectra reveals that they exhibit the same preedge and near-edge structures and shapes. This provides unequivocal evidence that the local coordination geometry and valence structure of Mn centers remains the same in the alloyed compounds and pristine β-Ga_2_O_3_.

### Temperature Dependence of the PL Efficiency

**Figure 12:**
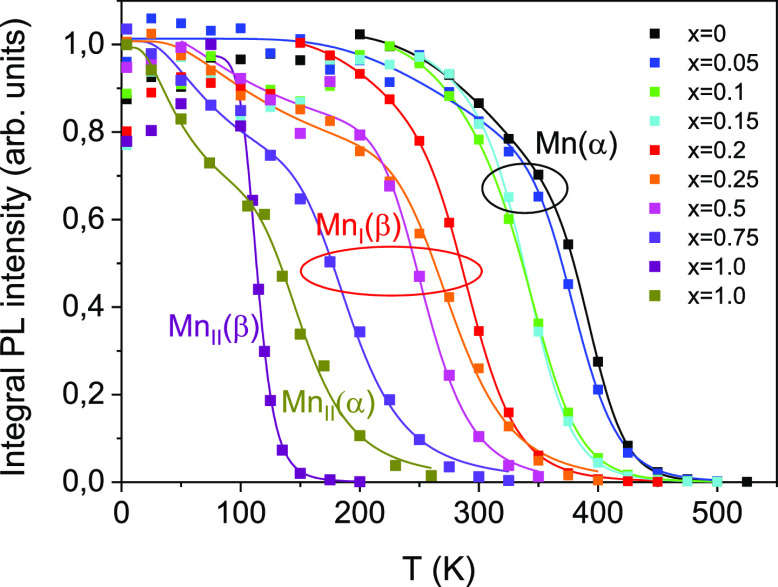
Normalized temperature dependencies of the Mn^4+^ emission intensity in the (Al_1–*x*_Ga_*x*_)_2_O_3_ solid solutions of different compositions and crystal structures. Solid lines represent fittings of the experimental points by eq 2 (see the text for details).

Temperature dependences of the Mn^4+^ emission intensity in the studied solid solutions are shown in Figure 12. Experimental data of the temperature dependence of the emission intensity (efficiency) are usually fitted by the equation
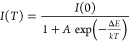
1where *I*(0) is the emission intensity at *T* = 0 K, Δ*E* is the activation energy of nonradiative transitions, and *A* is the ratio of the nonradiative transition probability to the radiative transition probability. However, it was found that the measured experimental data shown in Figure 12 cannot be adequately fitted by the model of the simple quenching process. Instead, it was shown that the temperature dependence of Mn^4+^ luminescence in the materials under study can be analyzed using a two-step quenching model given by following equation:^36^
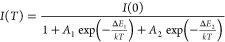
2where Δ*E*_1_ and Δ*E*_2_ are the activation energies of two competing nonradiative pathways with the corresponding parameters *A*_1_ and *A*_2_ related to the strength of the quenching processes. The use of eq 2 gives a better agreement of the theoretical curve with the experimental data, as shown in Figure 12. The energies Δ*E*_1_ and Δ*E*_2_ derived from the fitting are collated in Table 4. Here the uncertainties of the Δ*E*_1_ and Δ*E*_2_ energies were taken directly from the fitting as a fitting error. It should be mentioned that some of the compositions show a negative thermal quenching at low temperature (Figure 12). However, the low number of experimental points and their large spread in this low-temperature range do not allow us an accurate analysis in this temperature range using the approach proposed by Adachi.^37^ Therefore, thermal quenching starting only from the highest intensity (or starting from a plateau region) has been analyzed and fitted by us by eq 2. The results from Table 4 and Figure 12 show that the quenching temperature (*T*_1/2_) experiences gradual shifts toward lower temperatures as the Ga content increases. For the pressure-created α-Ga_2_O_3_:Mn^4+^, the quenching temperature is somewhat higher than that for β-Ga_2_O_3_:Mn^4+^. Both of the energies Δ*E*_1_ and Δ*E*_2_ tend to decrease with increasing Ga content. One of these energies can be interpreted as the energy distance from the minimum of the potential energy of the emitting ^2^E state to the level where it overlaps with the potential energy of the ^4^A_2_ ground state in the configurational coordinates. The other one can be ascribed to the energy distance from ^2^E to some defect states in the forbidden band gap of the host material, in line with that suggested in ref (37). A decrease of the last energy distance with an increase of the Ga content looks natural because the overall band gap of the host is strongly reduced from 8.8 eV for α-Al_2_O_3_ to about 4.8 eV for β-Ga_2_O_3_.^5^

**Table 4:** Parameters of Fits Obtained from the Temperature Dependence of the Emission Intensity (Equation 2) for Mn^4+^ Emission in (Al_1–*x*_Ga_*x*_)_2_O_3_

sample composition (*x* value)	Δ*E*_1_, meV	Δ*E*_2_, meV	*T*_1/2_,^a^ K
0	148 ± 7	1029 ± 6	380
0.05	91 ± 27	727 ± 210	370
0.1	190 ± 15	666 ± 40	336
0.15	186 ± 86	657 ± 89	336
0.2	90 ± 10	416 ± 17	285
0.25	16 ± 3	272 ± 24	262
0.5	14 ± 11	285 ± 50	247
0.75	11 ± 3	142 ± 17	174
1.0		147 ± 14	114
1.0^b^	7 ± 2	100 ± 12	132

a Temperature when the *I*(*T*) intensity is half of *I*(0).

b α-Ga_2_O_3_:Mn^4+^.

### Temperature Dependence of the PL Decay Time

**Figure 13:**
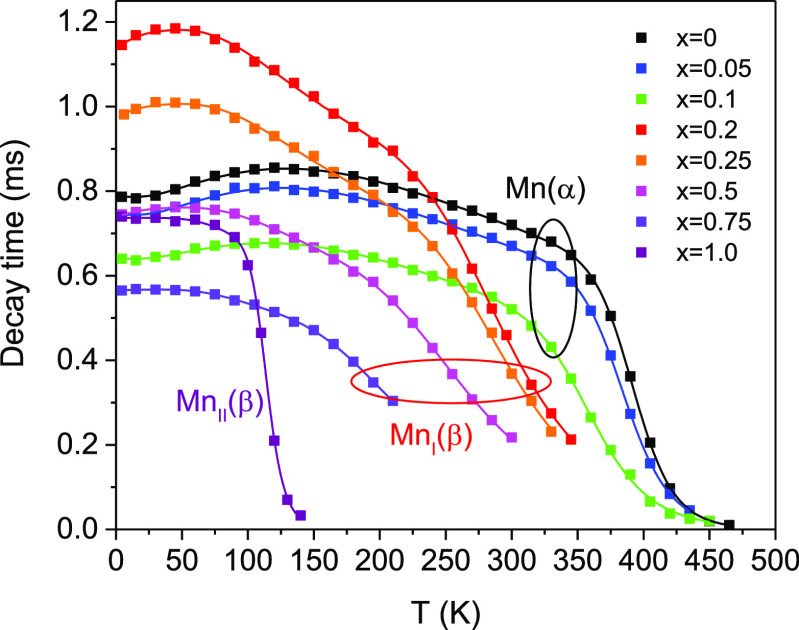
Temperature dependencies of the decay times of Mn^4+^ emission in the (Al_1–*x*_Ga_*x*_)_2_O_3_ solid solutions of different compositions. Solid lines represent fittings of the experimental points by eq 5.

The experimentally obtained temperature dependencies of the decay times of Mn^4+^ emission for the studied compounds are presented in Figure 13. The observed temperature dependencies can be explained by taking into account the main processes affecting the population of the emitting level ^2^E. A few early developed models allowed one to describe the major trends in the temperature dependence of the decay time of Mn^4+^.^38−40^ However, none of them were able to explain the rise of the decay time with heating observed at very low temperatures. This became possible with the introduction of a comprehensive model of the temperature dependence of the luminescence decay time in materials activated by transition metals developed recently by Mykhaylyk et al.^12^ The model accounts for the changes in the population of the emitting states due to the combined effect of thermally induced depopulation and phonon-assisted relaxation of the emission center. It should be accentuated that splitting of the ^2^E level included in this model is essential to explaining the initial rise of the decay time observed in the τ = *f*(*T*) curve. In this model, the radiative decay rate 1/τ(*T*) is derived as a weighted average of the rates from the individual levels:
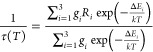
3where *R*_*i*_ are the radiative decay rates, *g*_*i*_ are the degeneracies of the states, and Δ*E*_*i*_ is the energy difference between the *i*th state and the lower excited level. It has been shown that a very good quantitative interpretation of the major features of τ = *f*(*T*) characteristics of Cr^3+^ and Mn^4+^ emission can be achieved by considering the thermalization process occurring between the *E̅* and 2*A̅* levels, phonon-assisted relaxation and depopulation of the levels due to thermally induced transitions from ^2^E to an upper state that promotes further quenching of the excited states,^12,23^ For this group of involved levels, the expression for the decay time constant is given as follows:
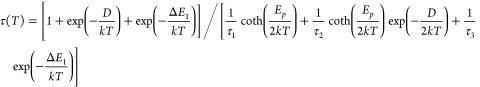
4

Here 1/τ_*i*_ (*i* = 1–3) are the radiative decay rates of the involved states, respectively, *k* is the Boltzmann constant, *D* is the energy split of the ^2^E levels, Δ*E*_1_ is the energy difference between the ^2^E and the upper state, and *E*_p_ stands for the “effective energy” of the phonons responsible for exchange with the sidebands.

It has been found that eq 4, however, is not adequate for fitting the temperature dependencies observed over a broader temperature range in the studied (Al_1–*x*_Ga_*x*_)_2_O_3_ solid solutions. The major deviation occurs in the region of thermal quenching of the emission where a rapid increase of the emission rate evidences the presence of an additional channel of nonradiative decay activated at high temperatures. It is worthwhile to note that the results of analysis of the temperature dependence of the emission intensity in materials under study give evidence of such an additional channel that requires activation energy Δ*E*_2_. We included this extra term in the model to find the relationship describing the kinetics of the system over a broad temperature range as follows:
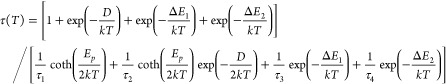
5

The formula was then used to fit the data obtained for the studied solid solutions. The results of the fitting shown in Figure 13 demonstrated good agreement of experiment and theory over the entire temperature range. The parameters of the fit are summarized in Table 5. It should be noted that, in the case of Ga_2_O_3_, this formula had to be modified to reflect changes in the scheme of emission transitions in the material and zero splitting for the ^2^E state of the Mn^4+^ ion.

**Table 5:** Parameters of Fits Obtained from the Temperature Dependence of the PL Decay Time (Equation 5) for Mn^4+^ Emission in (Al_1–*x*_Ga_*x*_)_2_O_3_

sample composition (*x* value)	τ_1_, ms	τ_2_, ms	*E*_p_, meV	*D*,^a^ meV	τ_3_, μs	Δ*E*_1_, meV	τ_4_, ms	Δ*E*_2_, meV
0	0.78 ± 0.02	1.13 ± 0.02	56.7 ± 1	10.0	0.1	225 ± 21	5 × 10^–13^	929 ± 46
0.05	0.74 ± 0.02	1.09 ± 0.02	54.4 ± 1	10.0	0.1	289 ± 20	2 × 10^–11^	784 ± 42
0.1	0.64 ± 0.02	0.80 ± 0.02	52.9 ± 2	10.0	0.1	189 ± 15	6 × 10^–9^	554 ± 15
0.15	0.64 ± 0.02	0.83 ± 0.02	56.9 ± 2	10.0	0.1	137 ± 5	2 × 10^–9^	557 ± 19
0.2	1.15 ± 0.01	1.24 ± 0.02	55.3 ± 2	2	0.7	32 ± 1	2 × 10^–5^	238 ± 5
0.25	0.98 ± 0.01	1.04 ± 0.02	50.9 ± 3	2	0.6	34 ± 1	2 × 10^–5^	245 ± 9
0.5	0.74 ± 0.01	0.79 ± 0.01	38.6 ± 3	2	0.57	35 ± 1	1 × 10^–5^	180 ± 20
0.75	0.56 ± 0.01	0.57 ± 0.01	35.0 ± 4	2	0.35	25 ± 2	4 × 10^–4^	124 ± 7
1.0	0.74 ± 0.01		27.1 ± 1			10 ± 4	1 × 10^–8^	178 ± 27

a The value of *D* is fixed to be equal to the energy splitting of the ^2^E level (as per the spectroscopic data).

**Figure 14:**
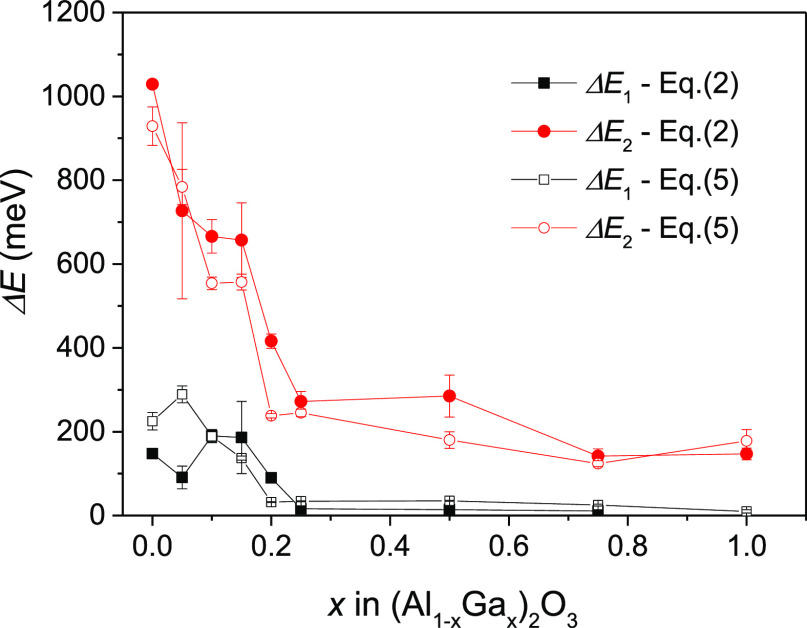
Comparison of the activation energies Δ*E*_1_ and Δ*E*_2_ determined from eqs 2 and 5.

Inspection of the fit parameters reveals a gradual decrease of the values of the activation energies Δ*E*_1_ and Δ*E*_2_ correlating with the trends for the activation energies obtained from the fitting the PL intensity versus temperature plots (Figure 14). The decrease of the effective phonon energy in Ga_2_O_3_-reach samples is consistent with an increase of the mass of Ga in comparison with Al, which leads to a reduction of the vibration frequencies of the bonds. Moreover, the value of *E*_p_ for pure Ga_2_O_3_ is very close to the dominant phonon mode of 29 meV in this material.^41^

### Thermometric Performance

The above results demonstrate efficient tuning of the temperature-dependent photoluminescence properties of the studied materials depending on the chemical composition. It is possible therefore to assume a utility of the materials for the luminescence thermometry around and below room temperature. The main interest is the temperature dependence of the decay time constant and the possibility of its tuning by changing the Al/Ga ratio in the Ga_2_O_3_–Al_2_O_3_ solid solutions.

**Figure 15:**
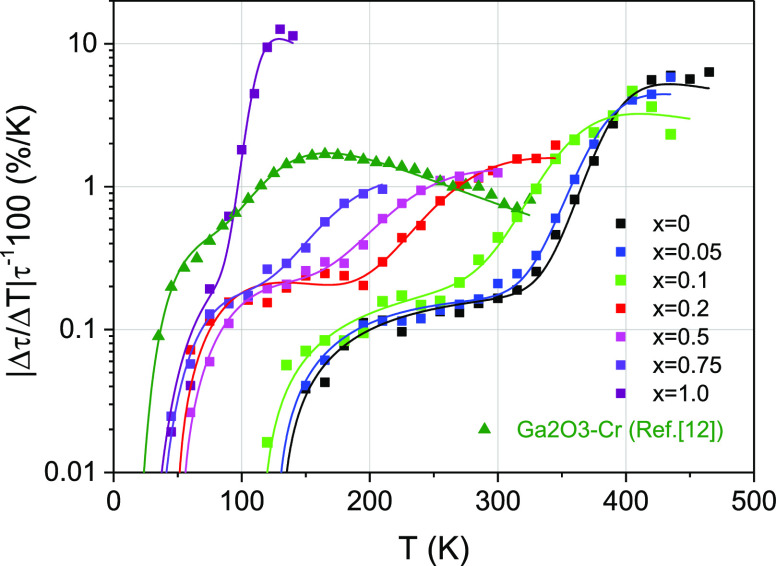
Specific sensitivity of the decay time luminescence thermometers based on the studied Mn^4+^-doped Ga_2_O_3_–Al_2_O_3_ solid solutions in comparison with β-Ga_2_O_3_:Cr^3+^.^12^ The solid lines were derived from the fitting using eq 5.

The performance of the studied materials for thermometry can be estimated using Figure 15, demonstrating the temperature dependencies of the specific sensitivity . As one can see from the figure, the α-(Al_1–*x*_Ga_*x*_)_2_O_3_:Mn^4+^ (*x* = 0, ..., 0.0.1) compounds have a maximal specific sensitivity of about 5%/K at 400 K, while the β-(Al_1–*x*_Ga_*x*_)_2_O_3_:Mn^4+^ (*x* = 0.2. . 0.0.5) compounds have a maximal sensitivity of about 1%/K near room temperature. β-Ga_2_O_3_:Mn^4+^ discovered in this work has the highest specific sensitivity of about 10%/K, even higher than that for β-Ga_2_O_3_:Cr^3+^,^12^ however in a narrow temperature range around 120 K.

## Conclusions

A series of microcrystalline phosphor materials with the nominal composition (Al_1–*x*_Ga_*x*_)_2_O_3_:Mn(0.05 atom %),Mg(0.05 atom %) with *x* = 0, 0.05, 0.10, 0.15, 0.2, 0.25, 0.5, 0.75, and 1.0 were synthesized. XRD analysis of the materials calcined at 1773 K revealed pure corundum and β-Ga_2_O_3_-type structures for the samples with *x* ≤ 0.05 and *x* ≥ 0.2, respectively, whereas the samples with intermediate compositions (*x* = 0.10 and 0.15) consisted of a mixture of these two phases in different proportions. The crystal lattice parameters, unit cell volumes, and average interatomic distances inside (Al/Ga)O_6_ octahedra increase systematically with increasing of Ga content in both the rhombohedral and monoclinic modifications of (Al_1–*x*_Ga_*x*_)_2_O_3_ alloys.

Photoluminescence measurements of Mn^4+^ also confirm the appearance of the monoclinic (β-type) phase of the ambient-pressure-synthesized (Al_1–*x*_Ga_*x*_)_2_O_3_ solid solutions because the Ga content is more than 10%. Depending on the chemical composition and crystal phase of the host material, different types of Mn^4+^ centers were revealed by low-temperature photoluminescence: (i) Mn occupying most probably Al sites in α-(Al_1–*x*_Ga_*x*_)_2_O_3_, marked as Mn(α), (ii) Mn occupying Al octahedral sites in β-(Al_1–*x*_Ga_*x*_)_2_O_3_, marked as Mn_I_(β), and (iii) Mn occupying Ga octahedral sites observed only in pure β-Ga_2_O_3_, marked as Mn_II_(β).

It was shown that photoluminescence features of the Mn(α) and Mn_I_(β) centers, like the temperature-dependent PL efficiency and PL decay time, can be gradually tuned in a wide range through modification of the Al/Ga ratio of the host lattice. In particular, the photoluminescence quenching temperature systematically shifts toward lower temperatures (from 380 to about 110 K) as the Ga content increases from 0 to 100%. Detailed analysis of the temperature dependencies of the PL efficiency and PL decay time allowed one to describe the main temperature-dependent processes affecting the population of the emitting level ^2^E.

Additionally, it was shown that the application of hydrostatic pressures of ≥19 GPa leads to the transformation of monoclinic β-Ga_2_O_3_:Mn^4+^ normally synthesized at ambient pressure to the rhombohedral corundum-like α-Ga_2_O_3_:Mn^4+^ with another emitting center, marked as Mn_II_(α).

Finally, it was shown that the temperature range of the maximal specific sensitivity of the decay time luminescence thermometers based on the studied Mn^4+^-doped Ga_2_O_3_–Al_2_O_3_ solid solutions can be effectively configured below room temperature by tuning the chemical composition of the host lattice. The obtained results indicate a high application potential of the studied materials for cryogenic luminescence thermometry. In particular, the highest specific sensitivity of about 10%/K at temperatures of around 120 K was observed for β-Ga_2_O_3_:Mn^4+^ examined in this work.
